# Twin and Sibling Studies Using Health Insurance Data: The Example of Attention Deficit/Hyperactivity Disorder (ADHD)

**DOI:** 10.1371/journal.pone.0062177

**Published:** 2013-04-24

**Authors:** Ingo Langner, Edeltraut Garbe, Tobias Banaschewski, Rafael T. Mikolajczyk

**Affiliations:** 1 Department of Clinical Epidemiology, Leibniz-Institute for Prevention Research and Epidemiology, BIPS GmbH, Bremen, Germany; 2 Department of Child and Adolescent Psychiatry and Psychotherapy, Central Institute of Mental Health, University of Heidelberg, Mannheim, Germany; 3 University of Bremen, Bremen, Germany; 4 Helmholtz Centre for Infection Research, Braunschweig, Germany; 5 Hannover Medical School, Hannover, Germany; Alexander Flemming Biomedical Sciences Research Center, Greece

## Abstract

**Background and Aims:**

Twin studies are used to assess the contribution of genetic factors to the aetiology of diseases. To show the feasibility of such research on the basis of health insurance data, we analysed twin and sibling data on the attention deficit/hyperactivity disorder (ADHD) in the German Pharmacoepidemiological Research Database (GePaRD).

**Methods:**

The GePaRD consists of data from four statutory health insurances, including around 17% of the total population of Germany. Among those insured in 2005, we identified 286,653 non-twin sibling pairs and 12,486 twin pairs. Each pair consisted of an index child (6 to 12 years old) and a co-sibling of equal age or up to five years older. ADHD cases were identified by hospital or ambulatory ICD-10 diagnoses (F90.0 or F90.1) and prescriptions. We estimated tetrachoric correlations, percentage of concordant pairs, concordance rates, and heritability. Weighted estimates for the indirect assessment of mono- and dizygotic pairs were derived.

**Results:**

Tetrachoric correlations were highest for twin pairs of the same sex (males: 0.85, 95% CI 0.81–0.89; females: 0.81, 95% CI 0.73–0.88) and lowest for opposite-sex non-twin sibling pairs (0.43, 95% CI 0.41–0.45). Heritability estimates were 0.88 (95% CI: 0.79–0.97) for males and 0.77 (95% CI: 0.60–0.95) for females.

**Conclusions:**

The study clearly reproduced the well-known strong genetic component in the aetiology of ADHD. This approach could be used for further assessments of genetic components in other diseases.

## Introduction

In epidemiological research, twin studies serve as a powerful method to assess the contribution of genetic factors to the etiology of diseases [1]. Generally, the recruitment of study participants for twin studies is either based on twin registries, and the selected twins are examined with respect to the outcome, or it is based on disease registries with subsequent identification of twin status and ascertainment of the disease in the co-twin. Both approaches require the additional collection of primary data. In Scandinavian countries where a life-long identification number is used, data from health services can be linked to twin registries allowing for studies without additional data collection [2]–[5].

Theoretically, health care data can be a source of information for twin studies, if the available data is sufficient to assess the twin status – for example by linking children with the same birth date to the same mother. There are several health care databases worldwide [6], [7], where such research was not conducted yet. We chose the example of the attention-deficit/hyperactivity disorder (ADHD) to assess the possibility to use health care data for a twin study in Germany.

Today, it is well accepted that the etiology of ADHD is influenced strongly by genetic risk factors [8]–[17] and most of the variation in the etiology and pathogenesis of ADHD is supposed to be attributable to genetic factors [8], [18]. The genetic influence on etiology is high regardless of whether ADHD is treated as a diagnostic category, a continuum of symptoms, or when using latent class analysis to define the phenotype [8]. Furthermore, similarity concerning ADHD between siblings appears to be solely a function of common genetic influences [14].

Twin studies based on primary data have been frequently conducted in the context of ADHD [8], [9], [19]. The aim of our study was to demonstrate the feasibility of twin and sibling studies in children and adolescents based on German health insurance data. An approach is presented to calculate separate estimates for monozygotic and dizygotic twins without direct knowledge of zygosity. The genetic component in the etiology of ADHD is assessed using within-pair similarity and heritability estimates.

## Materials and Methods

### Data Source

We used data from the German Pharmacoepidemiological Research Database (GePaRD). GePaRD was described elsewhere [20], [21], [22]. In brief, the database includes data of about 15 million insured persons representing about 17% of the total population of Germany. It consists of routinely collected reimbursement data from four statutory health insurance (SHI) companies and includes information about demographics, county of residence, as well as information about hospitalizations and ambulatory visits, procedures, and prescriptions. In addition to an individual identifier for each insured person there are family-IDs which link co-insured family members without an own income to the main insured person. For data protection reasons, only the year of birth of the insured persons and not the complete date of birth is available in GePaRD.

Prescription data is linked to a pharmaceutical reference database that contains detailed information on defined daily dose, dosage form, brand name, and package size.

All diagnoses, ambulatory as well as inpatient, are coded according to the German Modification of the International Classification of Diseases version 10 (ICD-10 GM). Inpatient diagnoses, dispensations of prescribed drugs and ambulatory services are recorded with an exact date, whereas ambulatory diagnoses can be related only to the quarter of the corresponding ambulatory visit.

Statutory health insurance is mandatory in Germany except for persons above a relatively high income threshold who are permitted to choose private health insurance instead. In total, about 90% of the German population is insured in one of several statutory health insurance companies (SHIs).

### Ethics Statement

The utilization of SHI data for scientific research is regulated by the Code of Social Law in Germany (SGB X). An ethical approval is not required as only routinely collected pseudonymized data are used. Privacy rights are guaranteed by a detailed data protection concept and do not require informed consent of the involved insurees. All involved SHIs, the Federal Ministry of Health (for data from SHIs active in multiple federal states) and the health authority of Bremen (for a SHI active only in the Federal State of Bremen) approved the use of the data for this study.

### Selection of Study Population

We selected data of children and adolescents who were between 6 to 17 years in 2005 and had an active continuous insurance period of at least 365 days beginning in 2005. This minimum insurance period was required for the ascertainment of ADHD cases (see below). Individuals were excluded if the information on sex or region of residence was missing. Using family-IDs, we identified children and adolescents from the same family. We assumed that they were full siblings, i.e. had the same father and mother. In the case of more than two siblings, we selected the youngest and the second youngest child from each family as a sibling pair. We further restricted the sample to those pairs in which the younger person was 6–12 years old and the maximum age difference between the siblings was five years. We assumed that siblings with the same year of birth were twins. The younger child of the non-twin pairs was denoted as the index child. In twin pairs the index child was randomly selected.

### Ascertainment of ADHD Cases

Ascertainment of ADHD cases was based on diagnoses and prescriptions reported for the insured persons in the SHIs data. Insured persons were ascertained as a ADHD case in three different ways: a) one inpatient diagnosis F90.0 or F90.1, or b) an outpatient F90.0 or F90.1 diagnosis and another outpatient diagnosis (F90.0, F90.1, or F90.9 in a different quarter or from a different doctor) within 365 days, or c) an outpatient F90.0 or F90.1 diagnosis and a methylphenidate or atomoxetine prescription within 365 days. At least one of these definitions had to be fulfilled. The index date for an ADHD case was defined as the date of the first event used for the case definition: either the date of hospitalization, the middle of the quarter of an outpatient ADHD diagnosis, or the date of a prescription. If more than one definition was applicable for a single person, the one with the earliest index date was chosen. We selected all cases with an index date in 2005. Each sibling was classified independently from its co-sibling.

### Inference of Zygosity Status

To allow conclusions about the genetic component in the etiology of ADHD, the within-pair phenotype similarity of siblings with a small genetic variance like in monozygotic (MZ) twin pairs and of siblings with a higher genetic variance like in dizygotic (DZ) twin or full-sibling pairs had to be compared. Within the study sample, MZ twin pairs could not be separated from DZ twin pairs with parity of sex ( = same-sex). Therefore, the number of concordant and discordant pairs within MZ twin pairs as well as within same-sex DZ twin pairs could not be directly identified but had to be estimated. We calculated the proportion (***p_ident_***) of monozygotic (MZ) twin pairs within the subgroup of same-sex twin pairs assuming that within the group of DZ twins the proportion of pairs with parity of sex ( = same-sex pairs) and the proportion of pairs with disparity of sex ( = opposite-sex pairs) is equal. Let *pairs_all_* be the number of all twin pairs and *pairs_opp_* the number of opposite-sex twin pairs then ***p_ident_*** is:
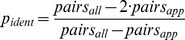
(1)


Since the same-sex DZ twin pairs have the same genetic within-pair similarity as same-sex non-twin pairs, we used the proportions of concordant and discordant pairs of the latter as weighting factors to calculate the respective numbers for concordant (*C*) and discordant (*D*) pairs for same-sex DZ and MZ twin pairs. This is expressed in the following equations:
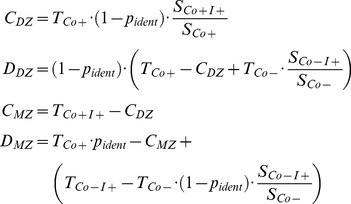
(2)


with :(included pairs have all parity of sex)
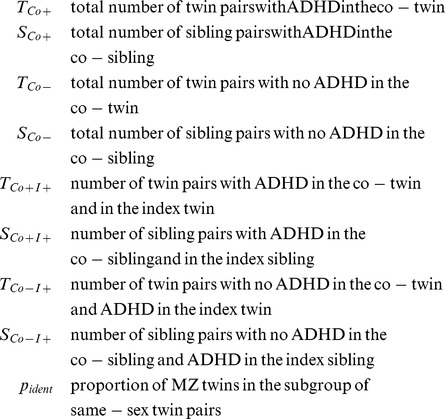
For same-sex pairs separate estimations were calculated for boys and girls.

### Statistical Analyses

We conducted descriptive analyses of the sex composition of the pair, the age difference within a pair, and the ADHD status of both siblings. For further analyses, we divided the sibling pairs into eight twin pair and eight non-twin pair groups based on all combinations of the three dichotomous variables ‘sex of the index child’ (male, female), ‘same sex within the pair (yes/no)’, and ‘ADHD in the co-sibling (yes/no)'. We calculated the proportion of concordant pairs concerning the ADHD-status and, following the method recommended by Newcombe and Altman [23], the corresponding 95% confidence intervals (CI).

To allow the comparison with the results of other twin studies, we calculated four traditional measures of within-pair similarity with regard to the ADHD status for the different subgroups of pairs: tetrachoric correlation, pairwise and probandwise concordance rates, and heritability. The tetrachoric correlation is a measure of association which is based on the assumption that the ordered, categorical variables have an underlying bivariate normal distribution [24]. Assuming that the ADHD status is the dichotomization of a continuous and normally distributed liability for ADHD, the tetrachoric correlation is the maximum likelihood estimator for the correlation coefficient in a bivariate normal distribution, when only information in the contingency table for the ADHD status is available. The pairwise concordance rate represents the proportion of affected pairs that are concordant. It is defined as C/(C+D), where C is the number of concordant pairs and D is the number of discordant pairs [25], [26]. For a group of twins in which at least one member of each pair is affected, the probandwise concordance is a measure of the proportion of twins who have the disease and who have an affected twin [27], [28]. It is defined as (2C_1_+ C_2_)/(2C_1_+ C_2_+ D) where C_1_ is the number of concordant pairs in which both twins of the pair have been ascertained (as ADHD cases) and C_2_ is the number of concordant pairs in which only one of the twins was ascertained and where the total number of concordant pairs is C = C_1_+ C_2_. For this proportion, corresponding 95% CIs were calculated [23]. Based on the tetrachoric correlations (*TC*) and their variances, sex specific heritability (*h^2^*) which is the proportion of variance of the phenotype that is determined genetically, was calculated by using Falconer’s formula h^2^ = 2(TC_MZ_ – TC_DZ_) [27], [29]. Corresponding 95% Wald CIs were calculated. Since age might affect ADHD diagnoses, we conducted a sensitivity analysis by restricting non-twin pairs to those with an intra-pair age difference of only one year.

All analyses were conducted using the statistical program package SAS version 8.2.

## Results

### Characteristics of the Study Population

**Figure 1:**
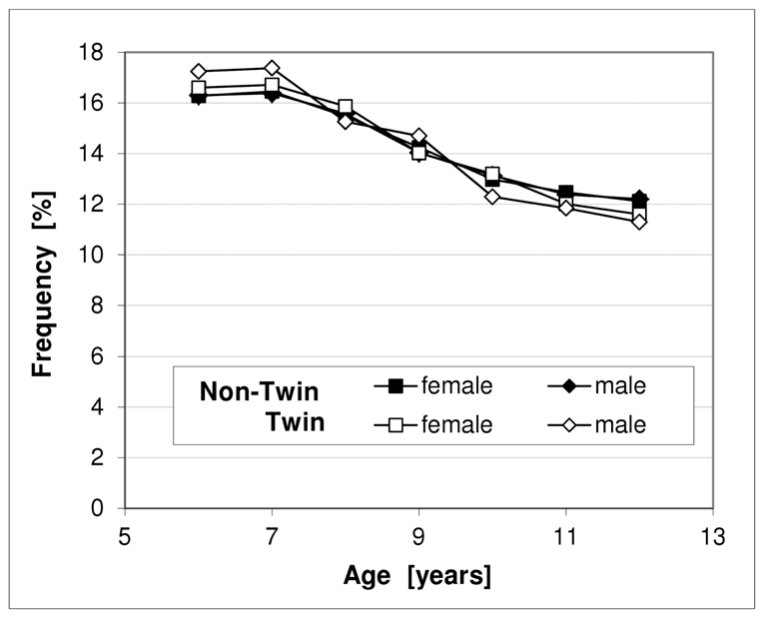
Age distribution of index children of twin and non-twin pairs by sex.

**Figure 2:**
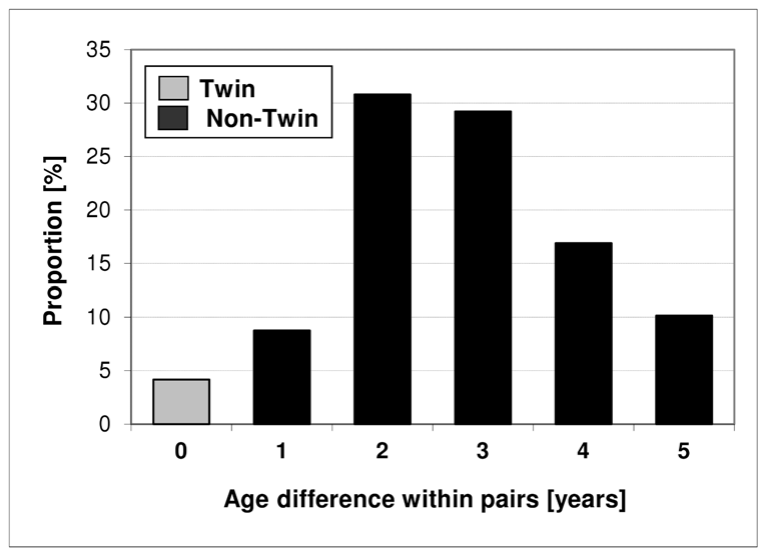
Age difference between the index child and the belonging co-sibling within.

Out of 1,615,822 children and adolescents in the age group 6–17, we identified 299,139 sibling pairs eligible for the study. In 12,486 sibling pairs (4.2% of all pairs) both siblings had the same year of birth and were assumed to be twins. Within this group, 64.2% of the pairs were same-sex pairs (32.0% male, 32.2% female) (Table 1). The proportion of males in all children of the non-twin pairs was 0.513, in all twins of same-sex twin pairs the proportion was 0.499. The relative age distribution was similar for twin and non-twin index children (Fig. 1). Most of the non-twin pairs showed an age difference of 2 or 3 years between the index child and its co-sibling (Fig. 2).

**Table 1:** Characteristics of the Sibling Pairs Selected out of Members of Four Statutory Health Insurance Companies.

Characteristics	Non-twin pairs		Twin pairs	
	N	%	N	%
**Sex of the index child**				
Male	147472	51.45	6233	49.92
Female	139181	48.55	6253	50.08
**Sex composition of the pair**				
Opposite-sex	144043	50.25	4473	35.82
Same-sex	142610	49.75	8013	64.18
**ADHD diagnosis in the index child in 2005**				
No	278265	97.07	12066	96.64
Yes	8388	2.93	420	3.36
**ADHD diagnosis in the co-twin or co-sibling in 2005**				
No	276944	96.61	12070	96.67
Yes	9709	3.39	416	3.33

In non-twin pairs, 2.9% of the index children and in twin pairs 3.4% of the index children fulfilled the ADHD case definition in 2005. In the non-twin pairs, the co-siblings showed a somewhat higher proportion of ADHD cases (3.4%) than the index children whereas in the twin pairs the proportion of ADHD cases differed only marginally between co-twins (3.3%) and index twins (Table 1). The distribution of definitions based on which ADHD subtype was ascertained as well as the proportion of cases with drug treatment within 365 days after first diagnosis were similar for index children of sibling pairs and in twins independently of index status (Table 2). In contrast, in co-siblings there were less ADHD cases ascertained by two ambulant diagnoses and more were ascertained by the combination of prescription and diagnosis. Also, a higher proportion of ADHD cases was drug treated within 365 days after initial diagnosis.

**Table 2:** Characteristics of ADHD Cases of Index Children and of Co-siblings of Sibling Pairs.

Characteristics	Non-twin pairs		Twin pairs	
	N	%	N	%
Index children with ADHS: type of earliest ADHD case definition				
All	8388	100.00	420	100.00
Hospital diagnosis	110	1.31	7	1.67
Two outpatient diagnoses	5170	61.64	247	58.81
Prescription+diagnosis	3108	37.05	166	39.52
Index children ever drug treated within 365 days after case date	4638	55.29	227	54.05
Co-siblings with ADHD: type of earliest ADHD case definition				
All	9709	100.00	416	100.00
Hospital diagnosis	112	1.15	9	2.16
Two outpatient diagnoses	5096	52.49	256	61.54
Prescription+diagnosis	4501	46.36	151	36.30
Co-siblings ever drug treated within 365 days after case date	6249	64.36	239	57.45

### Within-pair Similarity of ADHD

If the etiology of ADHD is genetically influenced, the presence of a co-sibling with ADHD should be a stronger risk factor for the index child in the case of identical genes (as for MZ twins) than in the case of lower gene concordance (in DZ twins and in full siblings on average 50%). Since the true zygosity status was not known, same-sex DZ twins and MZ twins were combined into one group. Based on equation (1), about 28% of all twin pairs and about 44% of all same-sex twin pairs were MZ twins. This resulted in a gene concordance for the same-sex twin pairs of 72% (on average).

Stratified by sex of the index child, proportions of pairs with concordant ADHD status within each pair are shown in Table 3. For same-sex twin pairs (i.e. DZ and MZ twins: M2, M6, F2, F6 in Table 3), we found significantly higher proportions of ADHD-concordant pairs than in the corresponding same-sex non-twin pairs (M1, M5, F1 and F5 in Table 3) whereas proportions of ADHD-concordant pairs for opposite-sex twins were similar to those in the corresponding non-twin PGs (i.e. M4 vs. M3, M8 vs. M7 and F4 vs. F3, F8 vs. F7 in Table 3).

**Table 3:** Frequency of Concordance Concerning ADHD Status in Sibling Pairs Stratified by Pair Characteristics.

Characteristics of pair group (PG)				Accordance of the ADHD status in a pair		% Concordant pairs (95% confidence interval)
PG No.	Type of pair	Sex composition of the pair	ADHD in the index child	Discordant [N]	Concordant[N]	
**Male index child**						
M1	Non-twin	Same	No	2956	68845	95.9 (95.7–96.0)
M2	Twins (DZ+MZ)	Same	No	87	3706	97.7 (97.2–98.1)
M3	Non-twin	Opposite	No	688	68474	99.0 (98.9–99.1)
M4	Twins (DZ only)	Opposite	No	15	2109	99.3 (98.8–99.6)
M5	Non-twin	Same	Yes	2293	984	30.0 (28.5–31.6)
M6	Twins (DZ+MZ)	Same	Yes	93	112	54.6 (47.8–61.3)
M7	Non-twin	Opposite	Yes	2899	333	10.3 (9.3–11.4)
M8	Twins (DZ only)	Opposite	Yes	94	17	15.3 (9.8–23.2)
**Female index child**						
F1	Non-twin	Same	No	804	65813	98.8 (98.7–98.9)
F2	Twins (DZ+MZ)	Same	No	32	3914	99.2 (98.9–99.4)
F3	Non-twin	Opposite	No	3377	67308	95.2 (95.1–95.4)
F4	Twins (DZ only)	Opposite	No	112	2091	94.9 (93.9–95.8)
F5	Non-twin	Same	Yes	747	168	18.4 (16.0–21.0)
F6	Twins (DZ+MZ)	Same	Yes	43	26	37.7 (27.2–49.5)
F7	Non-twin	Opposite	Yes	565	399	41.4 (38.3–44.5)
F8	Twins (DZ only)	Opposite	Yes	20	15	42.9 (28.0–59.1)

Abbreviations: ADHD, attention deficit/hyperactivity disorder; CI, confidence interval; DZ, dizygotic; MZ, monozygotic; PG, pair group.

The above results were confirmed by tetrachoric correlations (Table 4). Same-sex twins (DZ and MZ together) had a substantially higher correlation than opposite-sex twins or same-sex non-twins. In non-twins, opposite-sex pair groups had a lower correlation than the same-sex pair groups. In twins, this difference was more pronounced. Using equation (2), we estimated numbers for discordant and concordant pairs of MZ and same-sex DZ twins. Based on these numbers, we also calculated separate tetrachoric correlations for MZ twins resulting in quite high estimates for both sexes (Table 4: male 0.991 (95% CI: 0.983, 0.999), female 0.945 (95% CI: 0.899, 0.992)).

**Table 4:** Within-Pair Correlation with Respect to ADHD-Status for Twin and Non-Twin Sibling Pairs.

Characteristics of the Pair	Within-pair correlation (95% confidence interval)
Non-twin	
Male, same-sex	0.555 (0.535–0.574)
Female, same-sex	0.555 (0.517–0.592)
Opposite-sex	0.429 (0.408–0.449)
Twin	
Male, same-sex (DZ+MZ)	0.846 (0.806–0.886)
Female, same-sex (DZ+MZ)	0.805 (0.725–0.884)
Opposite-sex	0.502 (0.401–0.602)
DZ, male, same-sex ^a^	0.551 (0.437–0.666)
MZ, male ^a^	0.991 (0.982–0.999)
DZ, female, same-sex ^a^	0.555 (0.352–0.758)
MZ, female ^a^	0.942 (0.894–0.991)

Abbreviations: DZ, dizygotic twins; MZ, monozygotic twins.

Note: ^a^ Estimation of correlation and 95% confidence interval is based on counts calculated by using the formulas (2) presented in the methods section.

Contrary to the within-pair correlations which differed only marginally by sex for MZ twins and for DZ twins, the concordance rates showed somewhat higher values for male twin pairs than for female twin pairs (Table 5). However, overall the concordance rates for same-sex DZ and MZ twin pairs provided a similar picture of a strong genetic component as the within-pair correlations.

**Table 5:** Pairwise and Probandwise Concordance Rates for ADHD Status of Same-Sex Twin Pairs.

Type of twin pair	Concordance rate [%] ^a^			
	Pairwise	95% CI	Probandwise	95% CI
Male, dizygotic	15.5	(10.9–21.5)	26.8	(21.2–33.2)
Male, monozygotic	75.2	(66.4–82.2)	85.8	(80.2–90.0)
Female, dizygotic	9.7	(4.5–20.2)	17.8	(10.3–29.0)
Female, monozygotic	46.7	(32.7–61.2)	63.7	(51.4–74.4)

Abbreviations: CI, confidence interval.

Note: ^a^ Estimation of concordance rate and 95% confidence interval is based on counts calculated by using the formulas (2) presented in the methods section.

Based on the within-pair correlations of same-sex DZ pairs and MZ pairs, heritability was estimated at 0.88 (95% CI: 0.79, 0.97) for males and 0.77 (95% CI: 0.60, 0.95) for females.

In the sensitivity analysis restricting non-twin siblings to pairs with a maximum one-year difference in birth year, the results remained qualitatively unchanged (results not shown).

## Discussion

We demonstrated how traditional measures for a genetic component in the etiology of a disease can be estimated using a population-based health care database. The study sample included about 287 thousand non-twin sibling pairs and about 12.5 thousand twin pairs from all over Germany. To our knowledge this is the first study of this kind. We could reproduce previous qualitative and quantitative findings that postulated a strong genetic component in the etiology of ADHD [8], [10]–[17]. We presented an approach to calculate estimates for MZ and DZ twins without direct knowledge of the individual zygosity status. In our study, within-pair correlations were substantially higher for twin pair groups including MZ twins than for groups including DZ twins or non-twin siblings only, respectively. The lower correlations for opposite-sex non-twin pairs compared to same-sex non-twin pairs might indicate sex specific influences. Our within-pair similarity estimates for same-sex DZ twins and MZ twins as well as our heritability estimates were in line with published results of classical twin studies [8], [13], [18], [30]–[32]. These studies consistently demonstrated high heritability in the range of 60% up to nearly 100% and showed an average concordance rate of 0.76, indicating that genetic factors explain 70–80% of variation in ADHD [19]. The ratio of the correlation estimates for DZ and MZ twins (close to 2), found in our study, is consistent with findings presented by other authors [9], [12], [14] who inferred the lack of a major dominant genetic factor and a minor role of a shared environment in the etiology of ADHD from this result.

The main advantages of using electronic health care databases for the indirect assessment of genetic etiology of diseases are the large size and the population-based framework of study samples without additional primary data collection and the same availability of routinely recorded data for all study participants. In contrast to primary (field based) studies, the use of health care databases limits the potential for selection bias due to non-participation of invited study participants and recall bias, if past exposures are to be ascertained. Since all participants and especially both siblings of a pair are subject to the same case definition algorithm, and since the same type of data is used for all participants, further possible biases of primary studies like information bias or selective misclassification are excluded. Further, the large sample size allows fine stratification and comparison of subgroups. We were able to include over 12,000 twin pairs in the analysis, while past studies were based on 900 [33], 2,846 [34], 1,162 [12], or 3,853 [30] pairs. Additionally, we included about 287 thousand non-twin sibling pairs providing information about the generalizability of the results found for twins [12].

A limitation of the health care data is the fact that the family ID might not indicate a full sibling status. For example, some children could have been adopted in which case they would share less genetic material with their siblings leading to a downwards bias in the corresponding estimates. Unfortunately, there are no statistics on the percentage of full biological siblings in different age groups. Compared to the overall number of births, adoptions constitute a small share. Based on the numbers from the Federal Statistical Office, the ratio of adoptions to births continuously decreased from 1∶100 to 1∶145 between 1993 and 2005, which corresponds to the age group analysed in the current study. Apart from adoptions, patchwork families (with children from previous partnerships but not necessarily adopted) also contribute to the overall number of non-biological siblings, but there are no representative data quantifying this phenomenon for Germany. In patchwork families children are likely to be insured with their own biological parent and thus would not appear as siblings in our data if each parent had a separate insurance. Given the fact that we identified only siblings with the same main insured person, it is likely that in our dataset full biological siblings are overrepresented compared to the entire population.

Furthermore, due to data protection reasons, GePaRD only contains the year of birth. Children born at the beginning of a year can have a sibling with the same birth year. This could have led to a misclassification of some siblings as twins. However, based on data of a previous study, where we reconstructed the date of birth for newborns by building mother-child pairs [35], we found that 97.2% of siblings with the same year of birth were true twins. In an additional analysis including all children born in 1999 in our database, we obtained 16.1 twin births per 1000 births, which differs from the value of 14.9 given by the Federal Statistical Office for Germany. This slight overestimation of twins could be caused by a higher use of assisted reproduction techniques (ART) [36] in our sample. Using data of the German In Vitro Fertilization (IVF) Registry [37], twin births achieved by ART were estimated at 11.3% of all twin births in Germany from 1997 to 1999. Since insured persons from the middle socioeconomic class are overrepresented in health insurances contributing to GePaRD compared to the remaining statutory insurances of Germany [38], the use of ART could have been higher in our database than in the overall population. Another limitation of the family ID in German health care data is that it is only applicable to link siblings among children or adolescents as the linkage via a main insured person expires if the previously co-insured person starts to have own income. This restriction might not apply to other health care systems.

Electronic health care data do not contain information on zygosity of twins. Our approach to calculate estimates for MZ and DZ twins was based on two assumptions. First, we made the assumption that within DZ twin pairs the frequency of same-sex and opposite-sex pairs is equal. It is commonly accepted that the sex composition of a DZ twin pair depends on the probability of male and female births. Based on data from James [39] and Derom et al. [40], the calculated proportion of males was the same in singletons and in dizygotic twins (proportion of males = 0.514) whereas in monozygotic twins there is a slight excess of girls (proportion of males = 0.484) [41]. Therefore, within DZ twin pairs the expected proportion of opposite-sex pairs would be 0.5 (2 * 0.514 * (1–0.514)). The proportion of males in singletons as well as the proportion of males within same-sex twins (which is a mixture of dizygotic and monozygotic twins) in our study population were in good agreement with the values presented above. However, the proportion of males among the estimated MZ twin pairs was 0.462, which showed the expected excess of girls in MZ twins but was somewhat lower than the value of 0.484 given by Hall [41]. Second, we assumed that the proportions of concordant and discordant pairs with respect to ADHD-status were the same for same-sex DZ twin pairs as for same-sex non-twin pairs. This means that the fraction of full biological siblings would have to be the same in both groups and concerns shared environmental as well as genetic influences. Full biological non-twin siblings and DZ twins on average share the same proportion of identical genes. The equal environments assumption, that MZ twin pairs are no more likely to share the environmental factors that are etiologically relevant to the phenotype under study than DZ twin pairs, has been repeatedly tested and found to be valid for numerous phenotypes, including many mental disorders [42]. Our approach additionally required that the equal environments assumption is also true for non-twin siblings. A recent study which included twins and non-twin siblings, showed that, in as much as environmental influences on adolescent psychopathology are shared, they are affecting siblings of different ages to a similar extent as siblings with no age difference ( = twins) [12]. On the other hand, there is evidence that shared environment plays only a minor role in the etiology of ADHD [12], [14]. Our results support this as the within-pair correlations for same-sex DZ twin pairs are about half as strong as for MZ twin pairs. The difference between the correlations for these groups would have to be smaller than 50% to indicate the influence of a shared environment [14].

To calculate heritability estimates, our approach required the use of within-pair correlations of non-twin sibling groups as proxies for same-sex DZ twins. The prevalence of ADHD diagnoses depends on age [19], [22], [43] which could have caused the difference in the proportion of ADHD-affected between index children and co-siblings in non-twins. Further, with an increasing age difference between non-twin siblings, the probability of concordant pairs among them decreases. This could have led to an underestimation of the concordance rates and of the genetic effects for non-twin siblings and as a consequence to an overestimation of the estimates for MZ twins. To minimize this possible bias, we restricted the age difference within a pair to a maximum of five years. However, an additional sensitivity analysis based on pairs which were not exceeding a one-year age difference demonstrated similar results as for the whole sample.

### Conclusions

We demonstrated the feasibility of twin and sibling studies in children and adolescents based on German health insurance data. We calculated traditional measures of genetic association used in twin studies and reproduced results obtained in primary studies of genetic influence in the etiology of ADHD. This approach to study heredity based on health care databases can be used to investigate genetic components in the etiology of other diseases.
